# Study on the Driving Performance and Influencing Factors of Multi-Electrothermal Co-Actuation Devices Considering Application Environments

**DOI:** 10.3390/mi16060603

**Published:** 2025-05-22

**Authors:** Yujuan Tang, Zihao Guo, Yujiao Ding, Xinjie Wang

**Affiliations:** 1 School of Intelligent Science and Control Engineering, Jinling Institute of Technology, Nanjing 211169, China; zhguo020528@163.com; 2 School of Mechanical Engineering, Nanjing University of Science and Technology, Nanjing 210094, China; yujiaoding@njust.edu.cn (Y.D.); xjwang@njust.edu.cn (X.W.)

**Keywords:** electrothermal actuator, multi-electrothermal, co-actuation device, influencing factors, drive performance, simulation analysis

## Abstract

Electrothermal actuators, with their simple structure, small size, strong anti-interference ability, and easy integration, have emerged as a promising solution for micro-drive technology. However, deploying them in extreme environments, such as the fuze systems—which demand exceptional reliability under high mechanical overloads. In this study, a device based on multi-electrothermal co-actuation is designed for the fuze system of loitering munition. The overall structure and work principle of the multi-electrothermal co-actuation device is discussed. Considering application environments, the effect factors of V-beam numbers, air gap, type of contact surface, external load force, periodic voltage and gas damping on the output performance of the multi-electrothermal co-actuation device are systematically addressed via simulation and experimental method. Furthermore, the high overload resistance performance of the co-actuation device applied in loitering munition is studied. The results show that the proposed multi-electrothermal co-actuation device could operate stably under a high overload (12,000 g/73.79 μs) environment, fully meeting the demanding requirements of fuze system for loitering munition. In addition, this study identifies laser processing-induced thermal gradients and mechanical stresses as critical fabrication challenges. This study provides significant insights into the design and optimization of multi-electrothermal actuation systems for next-generation fuze applications, establishing a valuable framework for future development in this field.

## 1. Introduction

Micro-actuators convert other forms of energy into mechanical energy and are the main devices in the mechanism that accomplish the drive and enable the output of force or displacement [1]. In general, micro-actuators can be classified into electrostatic [2], piezoelectric [3,4], electromagnetic [5], electrothermal [6], and so on. Compared with electromagnetic, electrostatic, shape memory alloy, and piezoelectric actuators, electrothermal actuators have become one of the most important driving methods in the field of micro-actuation technology [7] and neurotechnology [8] due to the advantages of the simpler structure, smaller size, no interference from electrostatic or magnetic conditions, and better compatibility with integrated circuits.

In order to realize the function optimization from small displacements into larger ones, the electrothermal actuators are usually combined with the lever, pawl flywheel, and other displacement amplification mechanisms, which greatly enhances the drive performance and applicability of the device. Many research studies have explored various novel configurations of electrothermal actuators and the analysis or optimization of their driving performance. A novel V-shaped electrothermal actuator proposed by Shen et al. [9] using surface silicon technology achieved significant displacement at lower voltages within a shorter response time. Nguyen et al. [10] developed an electrothermal V-shaped actuator using a surface sputtering process. Under a voltage of 16 V, the displacement of the sputtering V-shaped actuator was twice that of the non-sputtering, enhancing the displacement output capability of electrothermal actuators under the same voltage conditions. Tecpoyotl et al. [11] designed a novel asymmetric Z-shaped electrothermal actuator. At a voltage of 2 V, the output force increased by 370.48%, while the displacement decreased by 29.8%, making it suitable for applications where a significant driving force is required, but displacement output is not a critical factor. Vargas et al. [12] studied an electrothermal actuator based on a rotary system, whose claw displacement of the actuator reached 21.2 μm, with a driving force of approximately 34.2 μN. Although this actuator offers significant advantages in miniaturization, the array-based layout of its driving beams results in a complex structure. A new in-plane parallel electrothermal actuator proposed by Yen et al. [13] can drive the lever to produce in-plane displacement of about 177 μm under 6 V and 115.32 mW.

In recent years, electrothermal actuators have been applied in the Safety and Arming Device (SAD) of the fuze system. In modern fuze safety systems, functional requirements not only mandate that SADs incorporate recoverable functionality but also demand that the miniaturization of fuze isolation mechanisms be achieved while maintaining a certain explosion isolation distance to ensure reliable blast containment. However, in the existing SADs, the isolation mechanisms based on micro-actuators still face limitations in terms of size, displacement, and recoverable function. Researchers have begun to explore new structures and drive technologies to achieve more significant outputs. Ostrow et al. [14] designed a pawl-driven flywheel MEMS safe and arming device. Under 250 Hz periodic voltage, the slider moves from the initial (safety state) to the specified (un-safety state) position in only 0.325 s, with an output displacement of 785 µm. A high-speed linear electrothermal actuator for SAD systems designed by Li et al. [15] consists of four V-shaped micro electrothermal actuators, four lever amplification structures, a slider, and a spring. The slider’s maximum displacement and output force were under 23 V and 5.27 mN. An environmental force sensing combined with a circuit-controlled MEMS fuze was developed by Wang et al. [16]. The environmental force-sensing inertial switch controls the state of the first fuze according to the impact load; the electrothermal actuator is connected to the slider and acts to limit the displacement of the slider. Hu et al. [17] designed a bidirectional large-displacement MEMS platform consisting of multiple V-shaped and U-shaped electrothermal actuators. However, this platform still has the disadvantages of a complex structure and excessive size.

In our previous published paper [18], we presented the co-actuation device based on multi-electrothermal actuators and established the mathematical model. In this paper, we focus on investigating the output performance and influencing factors of multi-electrothermal co-actuation devices in applied environmental SADs. Firstly, the electrical–thermal–mechanical coupled simulation model of the multi-electrothermal co-actuation device is established. Subsequently, the high overload resistance performance of the multi-electrothermal co-actuation device is investigated. In addition, the effect factors of V-beams umbers, air gap, type of contact surface, external load force, periodic voltage, and gas damping on the output performance of the multi-electrothermal co-actuation device are analyzed in detail via simulation and experimental method.

## 2. Overall Structure of Multi-Electrothermal Co-Actuation Device

**Figure 1:**
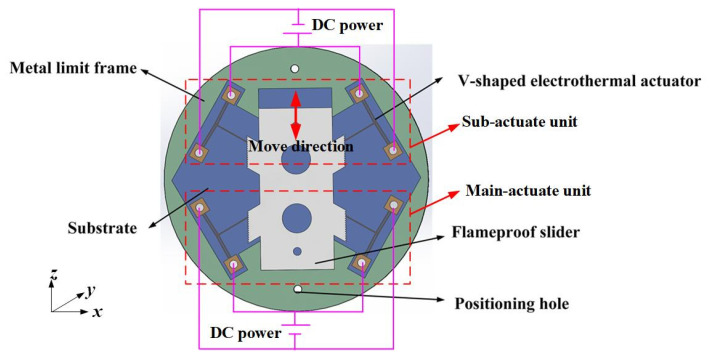
The structure diagram of the multi-electrothermal co-actuation device.

The overall structure of the multi-electrothermal co-actuation device is shown in Figure 1, which mainly includes the following: main-actuate unit (i.e., the two V-shaped electrothermal actuators responsible for the output displacement), sub-actuate unit (i.e., the two V-shaped electrothermal actuators responsible for the position-holding function), the substrate, the flameproof slider, the metal limit frame, and the upper and lower package covers. The V-shaped electrothermal actuator and the metal limit frame are fixed to the substrate through the positioning holes and fix screws.

**Figure 2:**
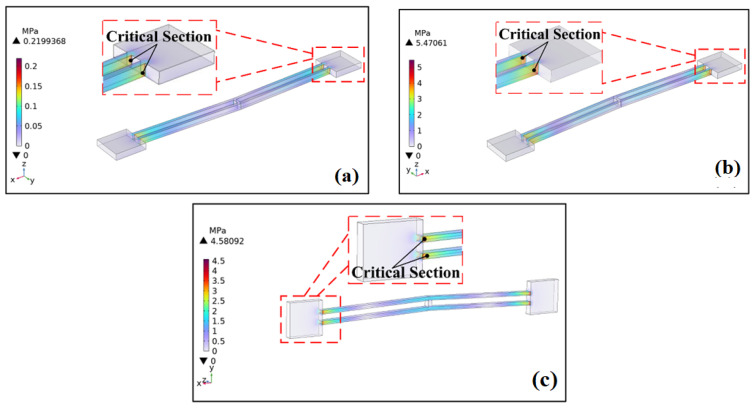
Stress distribution and critical sections under multi-directional impact loads: (**a**) ±*x* direction; (**b**) ±*y* direction; (**c**) ±*z* direction.

The V-shaped electrothermal actuator [19] consists of anchor points at both ends and a V-beam (cantilever beam), the structure of which is shown in Figure 2. The two ends of the V-beam are fixed to the substrate through anchor points to form a stable support.

When a voltage is applied across the anchor points, there is an electric current flowing through the V-beam and generating Joule heating within the beam structure. This heating causes thermal expansion of the electrothermal actuator. Due to the inclined angle of the V-beam, a non-uniform thermal stress field is induced during thermal expansion, resulting in bending deformation at the V-beam tips. This deformation is manifested as a linear output displacement and actuating force at the tip of the push lever, enabling the actuator to push or pull the target as required to meet the actuation demands. When the flameproof slider is required to output displacement in the +*z* direction, it is mainly actuated by the main-actuate unit. Conversely, when the slider needs to output displacement in the −*z* direction, it is mainly actuated by sub-actuate unit.

According to the miniaturization requirements of the fuze system for loitering munition, the final geometric parameters of the single V-shaped electrothermal actuator are provided in Table 1.

## 3. Analysis of High Overload Resistance Performance of the Multi-Electrothermal Co-Actuation Device

The multi-electrothermal co-actuation device studied in this paper is primarily designed to drive the isolation device of the SAD. As a vital driving part, it plays an essential role in initiating and controlling the fuze. It is required not only to output the target displacement to drive the flameproof slider but also to possess sufficient resistance to high overload conditions. The impact overload for the V-shaped electrothermal is primarily based on the acceleration generated by ammunition drops during the service phase, which is approximated as a half-sine function [20], given as follows:

(1) $$a\left(t\right)=\left\{\begin{array}{l} G\sin\left(\frac{\pi }{\Delta t}t\right)\begin{array}{cc} & \;\;\;\;\;t\leq \Delta t \end{array}\\ 0\begin{array}{cccc} & & & t>\Delta t \end{array} \end{array}\right.$$

where *G* is the overload acceleration amplitude, and Δt is the pulse width.

With an acceleration set to 10,000 g and a pulse width of 75 μs, the stress distribution of the actuator under impact loading applied in six directions—±*x*, ±*y*, and ±*z*—is shown in Figure 2.

The electrothermal actuator is made of stainless steel 304. If the maximum stress afforded by the electrothermal actuator is below the allowable stress of the material, [*σ*] = 386 MPa, the actuator can maintain regular operation. In all six directions, the maximum stress (i.e., at the critical section) occurs at the connection between the V-beam and the anchor point. Depending on the direction of the applied impact load, the critical section appears on different surfaces of the connection. The maximum stress at the critical section is about 0.0164 MPa, significantly lower than the allowable stress of the material.

Since the V-shaped electrothermal actuator is designed with a push lever, a structural transition exists at the connection between the push lever and the V-beam. In addition, the push lever is relatively long. Therefore, it is necessary to analyze whether the push lever deforms when subjected to high overload in the direction of the electrothermal actuator’s deformation (*x* direction).

**Figure 3:**
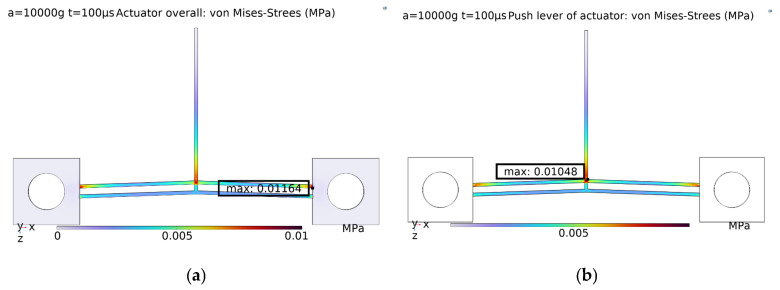
Stress distribution of the actuator under impact load in the *x* direction: (**a**) overall actuator; (**b**) push lever of the actuator.

Figure 3 compares the maximum stress at the actuator and the push lever under an impact load with a pulse width of 100 μs and an amplitude of 10,000 g. Under high-overload impact conditions, the maximum stress at the push lever is about 0.01048 MPa, significantly lower than the allowable stress of the material. Therefore, the push lever does not affect the location of the actuator’s critical section, which remains at the connection between the anchor and the V-beam.

**Figure 4:**
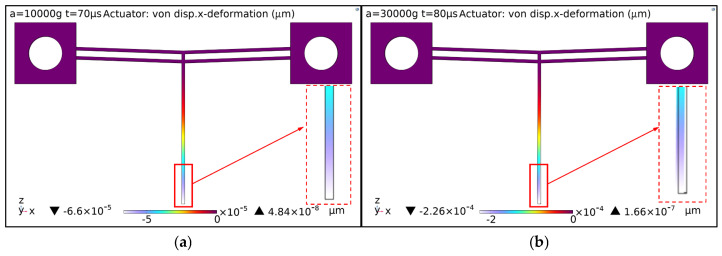
Deformation of the push lever under impact loads in the *x* direction: (**a**) 70 μs/10,000 g impact load; (**b**) 80 μs/30,000 g impact load.

Figure 4 shows the deformation of the push lever under impact loads in the *x* direction with varying pulse widths and amplitudes. Under an impact load with a pulse width of 70 μs and an amplitude of 10,000 g, the deformation of the push lever is minimal. When subjected to an impact load with a pulse width of 80 μs and an amplitude of 30,000 g, the push lever exhibits slight deformation.

The analysis results indicate that the metal-based V-shaped electrothermal actuator with a push lever can withstand high-overload impact loads with pulse widths ranging from 50 μs to 100 μs and amplitudes reaching tens of thousands of g. It can resist various impacts encountered by loitering munitions during the service phase without fracture or significant deformation, fully meeting the high-overload resistance requirements of the SAD of the loitering munition. In addition, simulation results demonstrate that the metal-based electrothermal actuator offers superior overload resistance performance compared to silicon-based electrothermal actuators [21].

## 4. Simulation Analysis of Performance-Influencing Factors of the Multi-Electrothermal Co-Actuation Device

### 4.1. Structural Influencing Factors

#### 4.1.1. Number of the Actuating V-Beams

**Figure 5:**
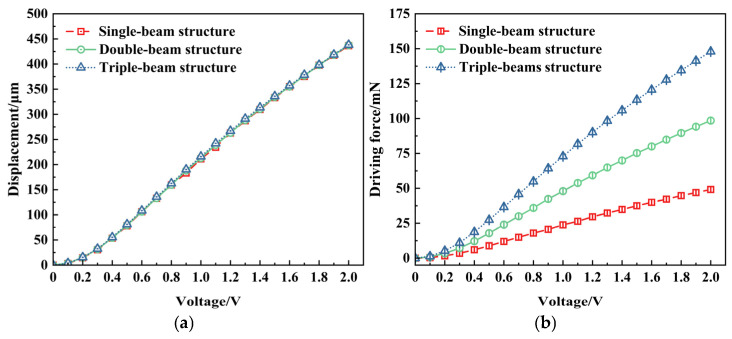
Influence of cascaded V-beams number on electrothermal actuator’s performance: (**a**) displacement variation; (**b**) driving force variation.

The V-beam is critical in actuation performance as the core component responsible for the electrothermal actuator’s output displacement. The number of cascaded V-beams directly affects both the output performance and structural stability of the V-shaped electrothermal actuator. Figure 5 shows the displacement and driving force variation under various voltages with different numbers of V-beams.

The results indicate that the number of V-beams has a relatively minor effect on the output displacement. However, under the same voltage, the driving force of the electrothermal actuator greatly increases with the increase in the number of V-beams.

Therefore, the number of V-beams can be selected when designing V-shaped electrothermal actuators based on specific application requirements. A single-beam or double-beam structure with the advantage of a simple structure is preferred for the applications where a high driving force is not essential. In contrast, a multi-beam cascaded structure can be adopted for applications requiring a higher driving force. The double-beam structure of the V-shaped electrothermal actuator is adopted in the following section of this paper.

#### 4.1.2. Air Gap

**Figure 6:**
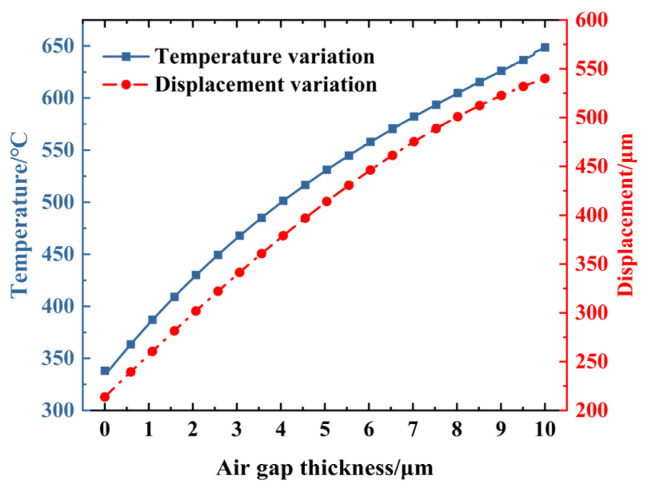
Influence of air gap thickness on V-shaped electrothermal actuator under 1.0 V.

As a thermal insulation layer, the air gap can effectively regulate the heat conduction efficiency between the electrothermal actuator and its surrounding environment. Herein, the air gap refers explicitly to the gap between the bottom of the electrothermal actuator and the substrate. The temperature and displacement of the electrothermal actuator vary with the thickness of this gap, shown in Figure 6.

At the same voltage, the electrothermal actuator’s temperature and displacement increase with the air gap’s enlargement. The reason is that the thermal conduction between the bottom of the actuator and the substrate is weakened as the air gap increases, which reduces heat dissipation through this path [22]. Consequently, more heat is retained within the electrothermal actuator, increasing internal temperature and a more significant output displacement. This effect has become more pronounced with larger air gap thicknesses.

In practical applications in SAD, the thickness of the air gap should be reasonably selected based on specific operating conditions. This ensures that the displacement is minimally affected by the friction between the bottom of the actuator and the substrate and helps to enhance the displacement under specific voltage or temperature constraints. Since the electrothermal actuator has a thickness of 200 μm and the flameproof slider has a thickness of 300 μm, an airgap of 100 μm is selected in the following section of this paper.

#### 4.1.3. Type of Contact Surface

**Figure 7:**
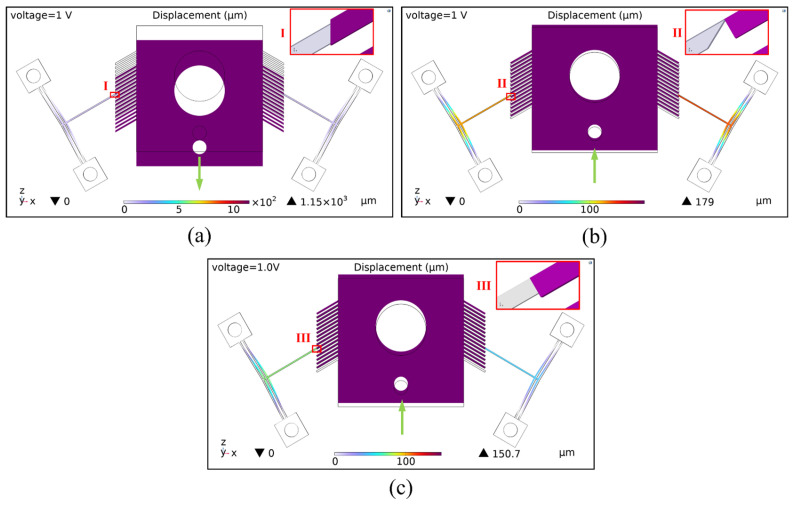
Displacement of the slider under different types of contact surfaces: (**a**) contact type I: vertical surface contact; (**b**) contact type II: point contact; (**c**) contact type III: inclined surface contact.

During the actuation process, the push lever of the actuator exerts an interaction force on the flameproof slider. Different types of contact surfaces result in different force distributions on the slider, thereby affecting its displacement. Finite element models were established for three contact surface types to analyze this effect. Figure 7 shows the simulation results of the slider’s displacement under each contact condition.

Figure 7a illustrates a “vertical surface contact” type, where the contact surface lies in the same plane as the slider’s displacement direction. Upon actuation, the actuator deforms within the x-o-z plane, but due to the minimal friction force along the *z* direction at the contact surface, it is insufficient to drive the slider upward, resulting in displacement of 0 μm.

Figure 7b shows a “point contact” type, in which the slanted tip of the push lever (with an inclined surface) contacts an oblique ratchet tooth (whose tooth surface forms an angle with the *z*-axis). Under a 1 V voltage, the slider achieves an upward displacement of about 179 μm along the +*z* direction. Although the contact surface is very small, the force transfer path is more direct in this contact type.

Figure 7c presents an “inclined surface contact” type, where the straight tip of the push lever (with a horizontal surface) contacts an oblique ratchet tooth (whose tooth surface forms an angle with the *z*-axis). Under the same voltage, the slider achieves a displacement of about 150.7 μm. However, the direction of force transfer in this contact surface may be easily shifted.

In summary, the contact type II configuration enables the slider to perform better displacement output. This will be further validated through experimental studies, fabricating and testing prototypes with different contact configurations.

### 4.2. External Influencing Factors

#### 4.2.1. External Load Force

**Figure 8:**
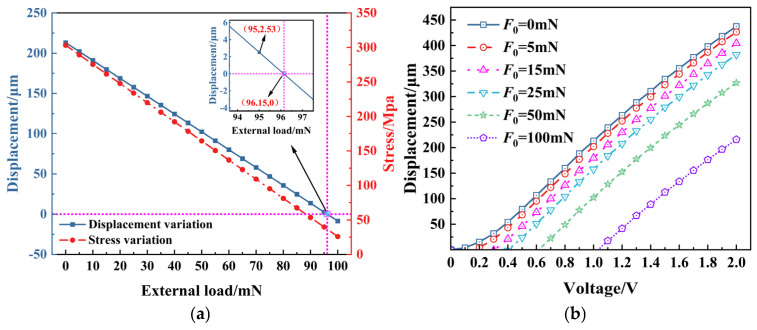
Influence of external loading on electrothermal actuator performance: (**a**) under 1 V; (**b**) under different voltages.

During the electrothermal actuator’s operation as a slider’s driving source, external load forces directly affect the actuator’s displacement performance. When the external load exceeds the maximum load capacity of the actuator, it may lead to irreversible deformation, malfunctioning, and reduced service life. Figure 8 shows the variation in the output displacement and the maximum thermal stress in the electrothermal actuator’s *y* direction (displacement direction) at a steady state under different external load forces.

As shown in Figure 8a, the maximum load that the V-shaped electrothermal actuator can withstand is approximately 96.15 mN. The actuator’s displacement and thermal stress exhibit a linear decreasing trend with the increase in external load. When the external load has become excessively large (though still within the material’s allowable stress capacity), the direction of the displacement and the thermal stress in the *y* direction may reverse, indicating that the electrothermal actuator has entered a failure state.

According to Figure 8b, the displacement of the actuator under a constant voltage gradually decreases with an increasing external load force. Under smaller loads, the displacement is primarily determined by the voltage, while at larger loads, the load force plays a dominant role.

Overall, in the design process of electrothermal actuators, it is essential to rigorously control the loading conditions and ensure that the applied load remains within the actuator’s maximum load capacity. For working environments where high external loads are unavoidable, the actuator’s load tolerance can be improved by increasing the driving voltage or optimizing geometric parameters—such as increasing the number of cascaded V-beams.

#### 4.2.2. Periodic Voltage

**Figure 9:**
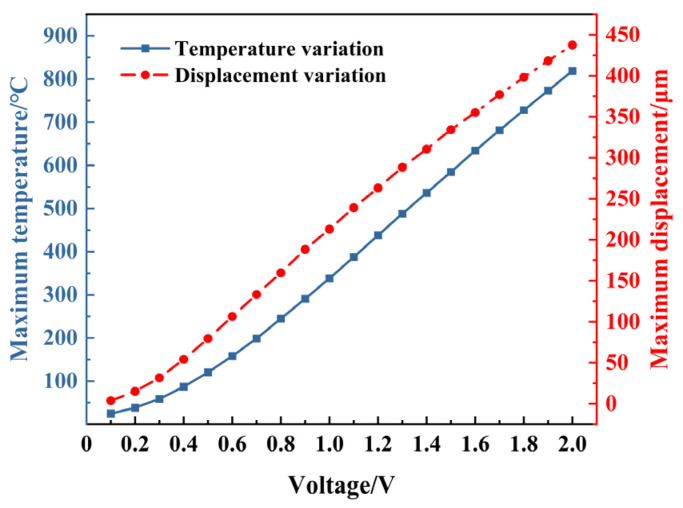
Variation in temperature and displacement with voltage amplitude.

From the perspective of energy flow [23], there is an electrical–thermal–mechanical energy conversion throughout the drive’s operation. Therefore, for the same actuator, the actuation performance ultimately depends on the amplitude of the voltage. However, for electrothermal actuators controlled by the periodic voltage to realize co-actuation driving, the frequency and phase of the periodic voltage may also have a different influence on the output performance. Figure 9 shows the variation curves of temperature and displacement with voltage amplitude for the electrothermal actuator in a steady state.

According to Figure 9, once the dimensions of electrothermal actuator are determined, the temperature and displacement with voltage in a steady state are synchronized with a nonlinear increasing trend as the voltage increases. The temperature should be within the safe range, and the voltage amplitude can be appropriately increased to obtain a more significant displacement.

The total duration of the periodic voltage and simulation calculation is given as follows:

(2) $$\left\{\begin{array}{l} V=\sin\left(2\pi ft+\varphi \right)\\ T_{0}=\frac{T}{4} \end{array}\right.$$

where *φ* and *f* are the phase and the frequency of the periodic voltage, respectively, and *T*_0_ is the total simulation time.

**Figure 10:**
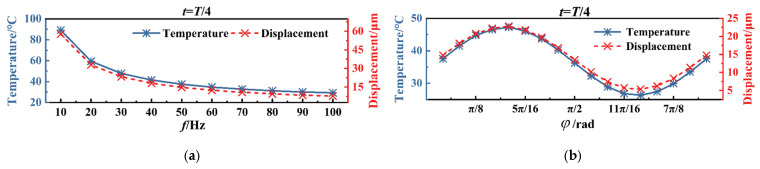
Influence of periodic voltage parameters on electrothermal actuator performance: (**a**) frequency variation, when *φ* = π/2; (**b**) phase variation, when *f* = 100 Hz.

When voltage signals applied to the same actuate unit vary in frequency (from 10 Hz to 100 Hz) and phase (from 0 to π), the temperature and displacement of the electrothermal actuator are shown in Figure 10. According to Figure 10a, the temperature and displacement of the actuator will decrease with an increase in the frequency. It can be seen from Figure 10b that both the displacement and temperature of the actuator exhibit sinusoidal variations with the phase.

As mentioned above, both the main-actuate unit and sub-actuate unit consist of one pair of V-shaped electrothermal actuators. When periodic voltages are applied to the two electrothermal actuators of the main-actuate unit or sub-actuate unit, the frequencies of the periodic voltages should be identical and the phases of the periodic voltages should be the same or have a phase difference of π/2, which ensures actuation synchronization among the actuate units and maintains the overall co-actuation of the device.

#### 4.2.3. Heat Transfer

Heat transfer is an important process during the operation of the multi-electrothermal actuator co-actuation device that directly affects the performance and reliability of the fuze system. An electrothermal actuator essentially functions as a resistive heating element, and in normal operation, the heat comes mainly from the joule heat generated internally.

**Figure 11:**
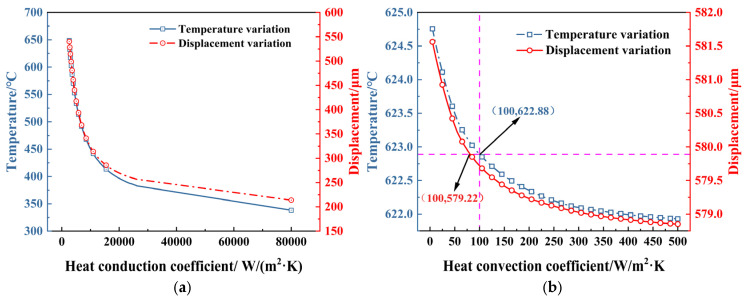
Influence of heat transfer on electrothermal actuator performance under 1.0 V: (**a**) heat conduction between the bottom of the actuator and the substrate; (**b**) heat convection between the surface of the actuator and the surrounding air.

The heat conduction coefficient between the bottom of the actuator and the substrate is defined as *S_t_*. In contrast, the heat convection coefficient between the surface of the actuator and the surrounding air is denoted as *h_c_*. If the voltage of 1.0 V is applied to the electrothermal actuator, the temperature and displacement vary with the heat conduction coefficient and heat convection coefficient, shown in Figure 11.

As shown in Figure 11a, when *S_t_* decreases, the heat dissipated through this part of the electrothermal actuator during the working process is reduced accordingly. As a result, the heat is retained more within the actuator, leading to a more significant displacement by the thermal expansion effect. Figure 11b shows that when *h_c_* increases, both the temperature and displacement of the actuator change within a small range, indicating that the effect caused by heat convection is small.

Under typical working conditions of electrothermal actuator applied in SAD, the absolute temperature ∆*T* of the actuator’s surface is generally around 300 °C~400 °C, and its thermal radiation power is much lower than that of high-temperature equipment (such as furnace walls, etc.). Assuming the absolute surface temperature of 400 °C, the heat radiation power is calculated as follows:

(3) $$E_{\varepsilon }=\varepsilon \sigma \Delta T^{4}=145.152\;{W/m}^{2}$$

where *σ* is the Stefan–Boltzmann constant, i.e., *σ* = 5.67 × 10^−8^ W·m^−2^·K^−4^, *ε* is the thermal emissivity, and the thermal emissivity of stainless-steel materials typically ranges from 0.1 to 0.3 [24].

Under the same conditions, when the heat convection coefficient *h_c_* is 70 W/m^2^·K, the heat dissipated through this part can be calculated as follows:

(4) $$q_{c}=h_{c}\Delta T=28,000\;{W/m}^{2}$$

**Figure 12:**
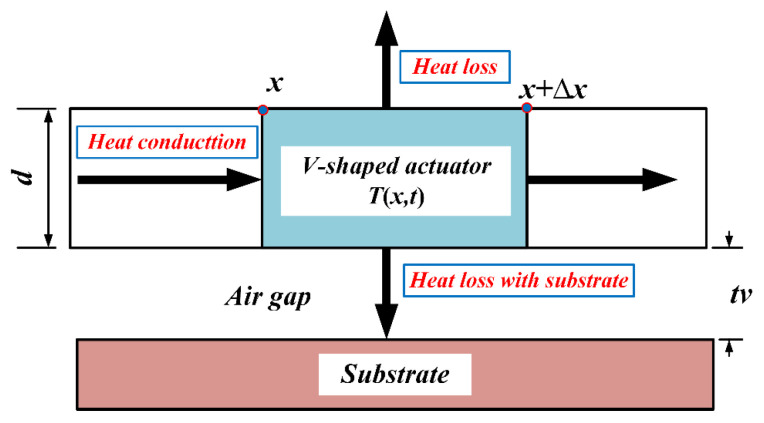
Schematic diagram of the heat conduction paths in the V-shaped electrothermal actuator.

In summary, heat loss due to heat radiation accounts for less than 0.5% compared to that caused by heat convection. Moreover, the influence of heat convection is much smaller than that of heat conduction. Therefore, heat conduction is the primary method of heat transfer in the operation of electrothermal actuators, which is consistent with the results in reference [25]. Therefore, the heat conduction paths of the V-shaped electrothermal actuator are illustrated in Figure 12.

#### 4.2.4. Gas Damping

The multi-electrothermal co-actuation device for SAD proposed in this paper is primarily influenced by sliding film damping during operation, while the effect of squeezing film damping is negligible. The film thickness is set to *t*_v_ = 100 μm, and the total duration is defined as *T*_1_ = 10 *T* = 20π/*ω*. A sinusoidal vibration velocity is applied to the actuator, expressed as

(5) $$u\left(t\right)=sin\left(\omega t\right)$$

**Figure 13:**
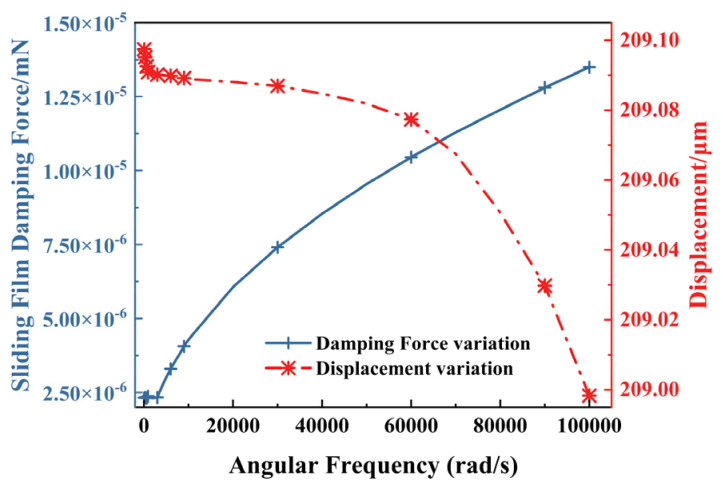
Influence of sliding film damping on electrothermal actuator performance.

Figure 13 shows the damping force and output displacement variation curves for the vibration of angular frequency. The damping force exhibits a clear nonlinear increasing trend with the increase in the angular frequency. However, the displacement remains stable from 200 μm to 210 μm. Even in high-frequency vibration conditions, the actuator maintains a minimum displacement of 200 μm. Therefore, the influence of damping on output performance can be considered negligible.

In addition, damping can be further optimized to ensure the stability of the fuze system at low frequencies and generate a sufficient response in a specific high-frequency range to prevent accidental triggering, thus improving the overall reliability of the fuze system.

## 5. Performance Experiments of the Multi-Electrothermal Co-Actuation Device

This section processes the prototype to verify the simulation analysis results, and the performance of the multi-electrothermal co-actuation device under the effect of different influencing factors is experimentally investigated.

### 5.1. Experiments of Processing Technology Influence on the Actuator Performance

**Figure 14:**
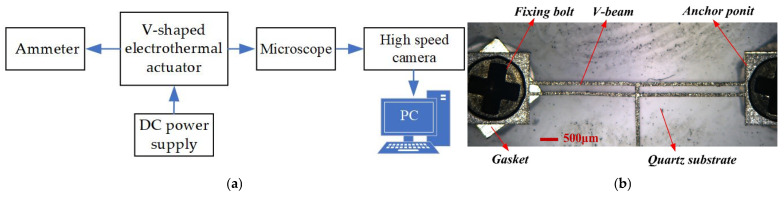
The experimental preparation: (**a**) the schematic block diagrams of experimental platform; (**b**) the prototype of the V-shaped electrothermal actuator.

As illustrated in Figure 14a, the experiment platform comprises a microscope, a DC power supply, an ammeter, a high-speed camera with a monitor, and dedicated measurement software. The V-shaped electrothermal actuator, fabricated using laser processing technology, is anchored to the substrate at both ends, as shown in Figure 14b.

In general, factors such as manufacturing techniques, processes, and tolerances affect the fabrication of the prototypes of the multi-electrothermal co-actuation device. In the case of laser processing technology, deviations in geometric parameters due to processing errors and various external factors during processing (such as temperature, external forces, etc.) may also influence the performance of the electrothermal actuator. This subsection analyzes the effects of temperature and external forces on the actuator’s performance during the laser fabrication process.

#### 5.1.1. Influence of Material Characteristics Changes on Actuator Performance

**Figure 15:**
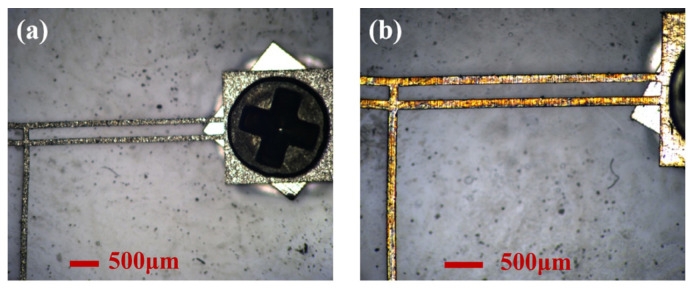
The prototypes of V-shaped electrothermal actuators: (**a**) before oxidation; (**b**) after oxidation.

The impact of excessive temperature during laser processing—caused by insufficient coolant or other factors—on the performance of the electrothermal actuator is investigated here. Such overheating may change the metal’s material properties or even oxidation. Figure 15 compares the actuator prototypes before and after oxidation.

**Figure 16:**
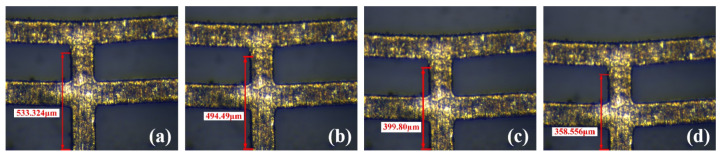
The deformation of the oxidized actuator under different voltages: (**a**) initial state; (**b**) under 0.5 V voltage; (**c**) under 1.0 V voltage; (**d**) under 1.2 V voltage.

**Figure 17:**
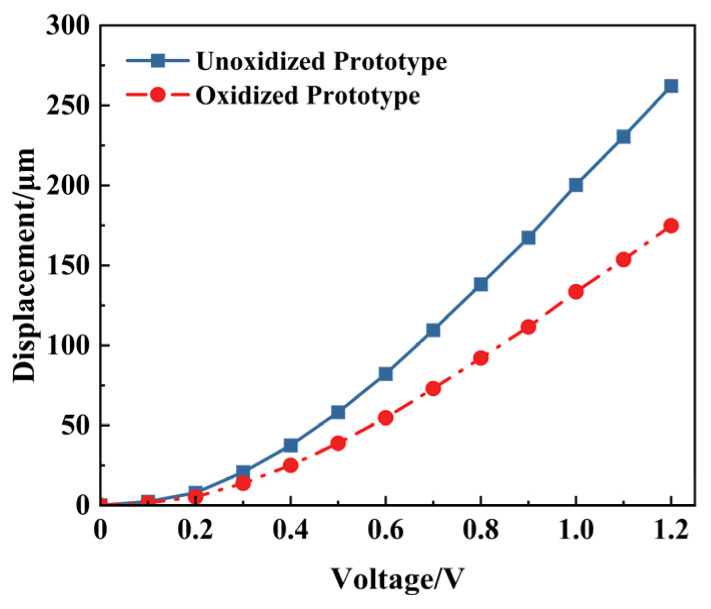
The experiment displacements of unoxidized and oxidized actuator prototypes.

Figure 16 shows the experimental results of the oxidized prototype in Figure 15b under different voltages. Figure 17 shows the displacement comparison between the unoxidized and oxidized prototype. The displacements of the actuator in the non-oxidized case at 0.5 V, 1.0 V, and 1.2 V are 38.834 μm, 133.524 μm, and 174.768 μm, respectively, which are significantly lower than those in the non-oxidized case under the same voltage. Oxidation of the material induced by the high temperature can change its coefficient of thermal expansion, which can adversely affect the actuator’s output performance.

#### 5.1.2. Influence of Deformation Caused by External Forces on Actuator Performance

**Figure 18:**
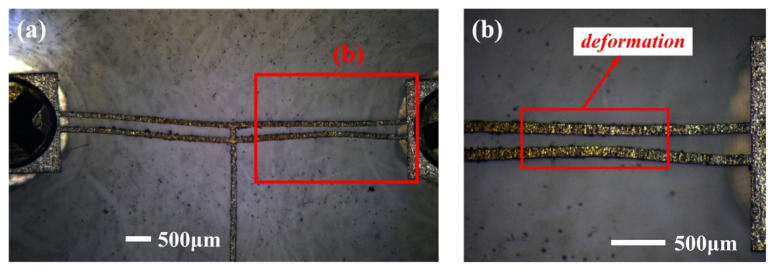
The prototypes of deformed V-shaped electrothermal actuators: (**a**) overall schematic; (**b**) detailed view.

**Figure 19:**
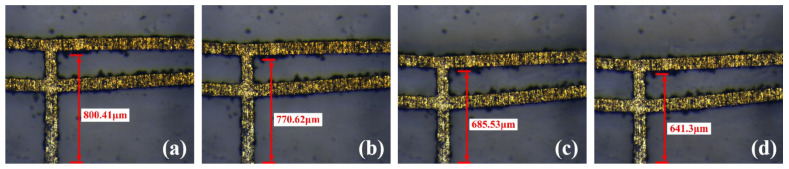
The displacements of the deformed actuator under different voltages: (**a**) initial state; (**b**) under 0.5 V voltage; (**c**) under 1.0 V voltage; (**d**) under 1.2 V voltage.

**Figure 20:**
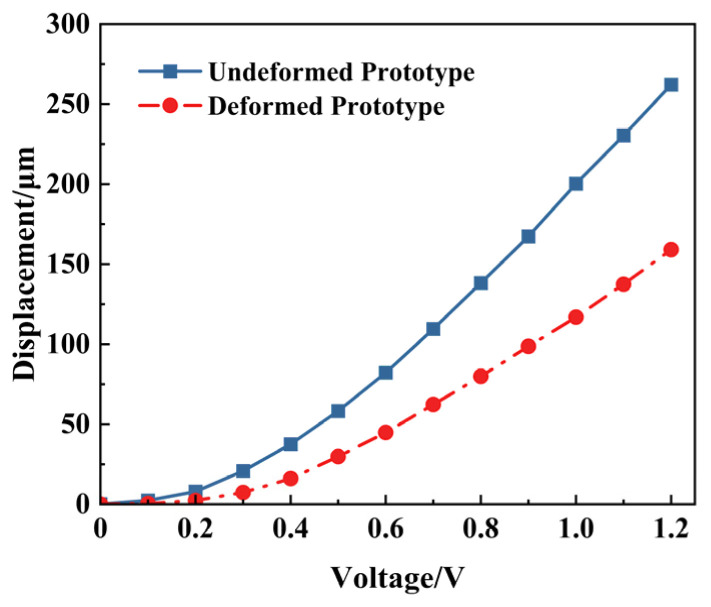
The experiment displacements of undeformed and deformed actuator prototypes.

The prototype could be irreversibly deformed on the V-beam due to external force before use, as shown in Figure 18. The displacements of this deformed prototype under different voltages are tested and shown in Figure 19. The corresponding displacements of the deformed actuator under 0.5 V, 1.0 V, and 1.2 V are 29.79 μm, 114.88 μm, and 159.11 μm, respectively. Furthermore, the displacement comparison between the undeformed and deformed V-shaped beams is shown in Figure 20. According to Figure 18, Figure 19 and Figure 20, under the same voltage, the displacement of the deformed prototype is reduced by at least 50% compared to the undeformed one. The deformation that occurs on the V-beam structure adversely affects its output, leading to a reduction in its output displacement.

From the above experimental results, both high temperature and external force will inhibit the output performance of the electrothermal actuator. Therefore, such influencing factors should be avoided as much as possible in the fabrication process to restore the ideal model and improve the reliability of the experimental results.

### 5.2. Experiment of Contact Surface Type Influence on the Slider Output Performance

The three prototypes corresponding to the simulation analysis in Section 4.1.3 were fabricated and numbered prototypes 1, 2, and 3, respectively, representing contact types I, II, and III.

**Figure 21:**
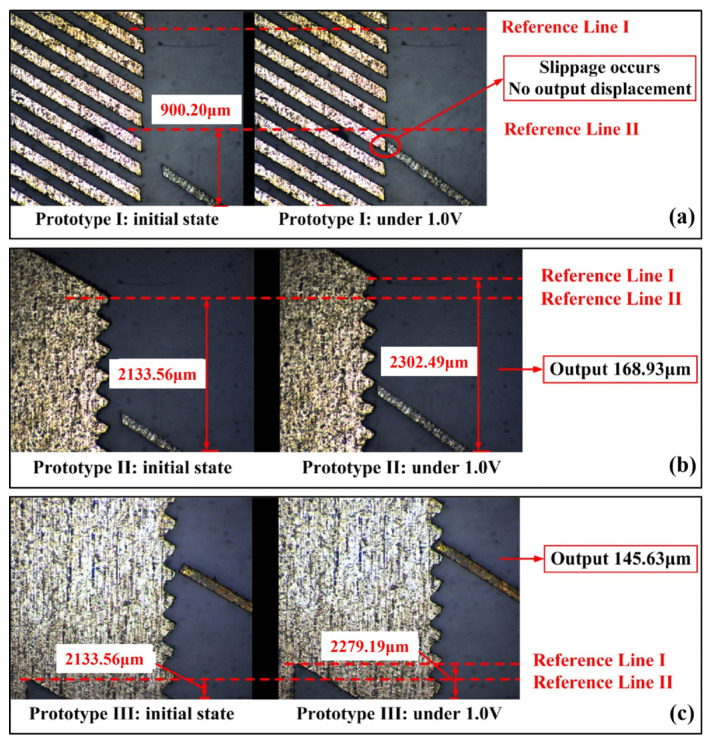
The slider displacement under different types of contact surfaces: (**a**) contact type I: vertical surface contact; (**b**) contact type II: point contact; (**c**) contact type III: inclined surface contact.

Figure 21 shows the slider’s displacement of different contact surface types when 1 V voltage is simultaneously applied to the main-actuate unit.

For prototype I, the displacement of the slider is almost 0 μm. Under the condition of prototype II, the slider outputs a displacement of about 168.93 μm when applying the voltage of 1.0 V, and under the same voltage, the slider in prototype III outputs a displacement of about 145.63 μm. This is consistent with the simulation results in Section 4.1.3, which verify the accuracy of the simulation analysis and the reasonability of choosing type II as the final contact surface of the device.

### 5.3. Experiment of High Overload Resistance Performance

**Figure 22:**
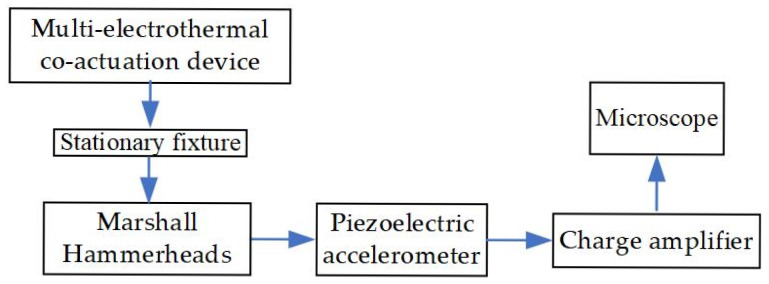
The Marshall hammer impact test platform.

The high overload resistance test is mainly accomplished with the help of the Marshall hammer impact test platform, which mainly consists of Marshall hammerheads, a piezoelectric accelerometer (CA-YD-111), a charge amplifier (YE5854A), and an oscilloscope, as shown in Figure 22.

Since loitering munition experiences relatively low launch overloads, the loitering munition is subjected to impact loads with pulse widths of 50 μs~500 μs and amplitudes of 5000 g~30,000 g, when it falls on hard targets such as concrete, steel plates, and cast steel during the loitering munition’s service phase. Herein, the high overload resistance of the co-actuation device under the impact loads with pulse widths of 70 μs~80 μs and amplitudes of 6000 g~9000 g are tested in the next section.

#### 5.3.1. Experiment Under Impact Load in Six Directions

Three impact loads are applied in six directions: ±*x*, ±*y*, ±*z*; the conditions of the prototype after impact, focusing on whether fractures or other damages occur at the critical sections of the prototype, are observed. The specific data of each impact load and the state of the prototype after impact are recorded in Table 2. The maximum impact loads in the six directions of ±*x*, ±*y*, ±*z* are 10,600 g, 11,000 g, 12,000 g, 10,600 g, 12,200 g, and 12,600 g. It can withstand an impact load of more than 10,000 g without fracture.

#### 5.3.2. Experiment Under Impact Load at an Angle of 30° to the *y* Direction

The V-shaped electrothermal actuator in the co-actuation device is arranged at an inclined angle of 60° to the displacement direction (*y* direction) of the flameproof slider, while the deformation direction of the V-beam is at an angle of 30° to the displacement direction of the slider. Eight impact tests were carried out, and the deformation or fracture of the actuator was assessed when the prototype was subjected to an impact load at an angle of 30° to the y direction, as shown in Table 3.

When the electrothermal actuator is subjected to an impact load with a pulse width of 73.79 μs and an amplitude of 12,000 g, it is hardly deformed, while at an impact load with a pulse width of 67.79 μs and an amplitude of 13,800 g, the push lever of the actuator undergoes a slight deformation, which can be observed through a microscope.

**Figure 23:**
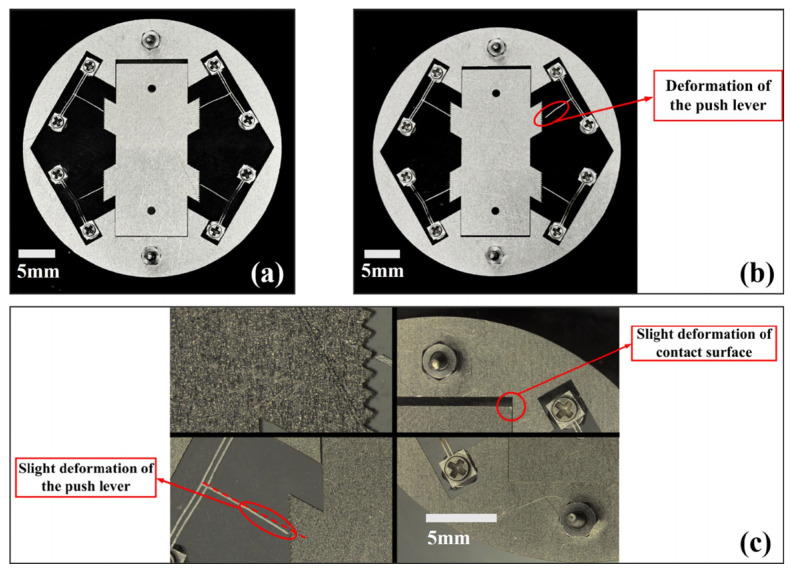
Comparison of the prototype before and after impact load 8 (13,800 g/67.79μs): (**a**) before impact load; (**b**) after impact load 8; (**c**) detailed view.

Figure 23 shows the state of the prototype before and after being subjected to impact load 8. The overall structure of the prototype remained intact after impact load 8, with no fracture of the slider, the V-beam, the anchor points, the push lever, or the connection between the anchor points and the V-beam (critical section). Some of the push levers of the electrothermal actuators showed a slight deformation, which could be observed through a microscope. Moreover, this deformation is within the tolerance of the material. The experimental results show that the device has excellent high overload resistance performance and strong bending resistance ability.

**Figure 24:**
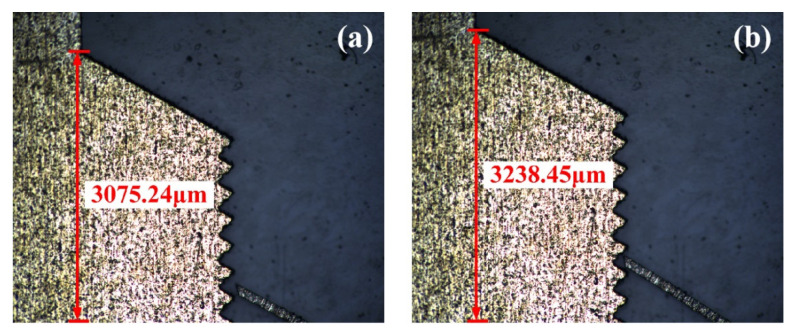
Displacement of the prototype after impact load 6 (12,000 g/73.79 μs): (**a**) initial state; (**b**) under 1.2 V voltage.

As shown in Figure 24, after being subjected to impact load 6, the slider achieved a displacement of 163.21 μm per step under 1.2 V. The prototype’s actuation performance was almost unaffected by the impact load.

The above experimental results verified that the co-actuation device studied in this paper has excellent high overload resistance performance and bending deformation ability. It can resist various high overload impacts of loitering munition in the service phase and meets the requirements of high stability and reliability of the device under the application of the fuze system.

## 6. Conclusions

This study focuses on the overall structure, performance simulation analysis, and experimental verification of the multi-electrothermal co-actuation device, particularly emphasizing its application value in the fuze safety system of loitering munition. The main research conclusions of this paper are listed as follows:

1. Based on finite element simulation, the effects of influence factors such as the number of V-beams, air gaps, contact surface type, external load force, periodic voltage, heat transfer, and slip film damping on the multi-electrothermal co-actuation device’s performance are investigated. The simulation results indicate that adequately optimizing structural parameters and driving conditions can significantly enhance the device’s performance. In addition, the flameproof slider’s contact surface II structure provides the best output displacement performance.
2. The multi-electrothermal co-actuation device has excellent high overload resistance performance. It maintains stable driving performance under high-impact load conditions, with only slight and recoverable deformation observed at the push lever, which meets the high overload resistance requirements of fuze applications.
3. Temperature changes and external forces during the laser fabrication process will influence the actuator’s performance to different degrees. Experimental results show that changes in the material properties of the structural deformation lead to performance degradation, so strict control of influence factors in the fabrication process is necessary to ensure the stability of the driving performance.

## Figures and Tables

**Table 1:** The parameters of the single V-shaped electrothermal actuator.

Parameter Name	Parameters	Physical Unit	Specific Value
Length of the actuator	*L_z_*	μm	7000
Thickness of the actuator	*d*	μm	200
Width of the actuator	*a*	μm	100
Tilt angle of the V beam	*θ*	°	2
Length of the lever	*L_p_*	μm	5000

**Table 2:** The experimental results under impact loading in six directions.

Prototype	Direction	Amplitude/g	Pulse Width/μs	Break or Not
Prototype 1	+*x*	8600/10,600/9200	71.79/91.79/73.29	Not
Prototype 2	−*x*	8400/9600/11,000	75.79/89.79/77.79	Not
Prototype 3	+*y*	7200/9600/12,000	79.79/75.79/73.79	Not
Prototype 4	−*y*	6400/10,600/9400	81.79/77.79/75.79	Not
Prototype 5	+*z*	8600/10,600/12,200	77.79/89.79/81.79	Not
Prototype 6	−*z*	7800/8400/12,600	71.79/71.79/75.79	Not

**Table 3:** The experimental results under impact loading at an angle of 30° to the *y* direction.

Impact Load Number	Amplitude/g	Pulse Width/μs	Deformity or Not
Impact load 1	7800	71.79	Not
Impact load 2	8600	75.79	Not
Impact load 3	9200	73.29	Not
Impact load 4	10,600	77.79	Not
Impact load 5	11,000	77.79	Not
Impact load 6	12,000	73.79	Not
Impact load 7	12,200	69.79	Not
Impact load 8	13,800	67.79	A little

## Data Availability

All data are presented within the paper.
